# Cellular Uptake Behaviors of Rigidity-Tunable Dendrimers

**DOI:** 10.3390/pharmaceutics10030099

**Published:** 2018-07-19

**Authors:** Hui Liu, Jingjing Wang, Wenchao Li, Jie Hu, Min Wang, Yuejun Kang

**Affiliations:** 1 Institute for Clean Energy and Advanced Materials, Faculty of Materials and Energy, Southwest University, Chongqing 400715, China; jingjingwang0502@gmail.com (J.W.); m13102303899@163.com (W.L.); 18284876279@163.com (J.H.); minwang@swu.edu.cn (M.W.); yjkang@swu.edu.cn (Y.K.); 2 Chongqing Engineering Research Center for Micro-Nano Biomedical Materials and Devices, Chongqing 400715, China

**Keywords:** PAMAM dendrimers, gold nanoparticles, rigidity, cellular uptake

## Abstract

Understanding of the interaction between cells and nanoparticles (NPs) is critical. Despite numerous attempts to understand the effect of several parameters of NPs on their cellular uptake behaviors, such as size, shape, surface chemistry, etc., limited information is available regarding NP rigidity. Herein, we investigate the effect of rigidity on cellular uptake behaviors of NPs, using generation 5 poly(amidoamine) dendrimer as a model. By harnessing the abundant inner cavity, their rigidity could be effectively regulated by forming size-tunable gold NPs. The NPs thus formed were well characterized and displayed similar hydrodynamic size, surface potential, fluorescence intensity, and distinct rigidity (owing to differences in the size of the Au core). Flow cytometry analysis revealed a positive correlation between NP rigidity and cellular uptake of NPs. Confocal microscopic evaluation revealed that the entrapped gold NPs may affect the intracellular localization of the internalized dendrimers. The present findings can potentially guide the preparation of suitable NPs for biomedical applications.

## 1. Introduction

The design and synthesis of desirable nanoparticles (NPs) is an important task for developing efficient carriers in the biomedical field. Till now, NPs with distinct physicochemical properties including size, shape, composition, surface potential, and surface chemistry have been developed for various biomedical applications [1,2,3,4,5,6,7,8,9,10]. When using nanomaterials in the biomedical field, it is critical to understand their interaction with cells [11,12,13]. The interaction between NPs and cells varies depending on their aforementioned properties [14,15,16,17,18]. However, limited information is available regarding the effect of NP rigidity on its interaction with cells [19,20,21,22]. Recently, Jiang et al. developed a class of novel polymeric core–lipid shell NPs with tunable rigidity to investigate their cellular uptake behaviors. Based on both experimental data and molecular dynamics simulations, the cell lines used in their study exhibited preferable intracellular uptake of NPs with higher rigidity [23].

Inspired by the prior work of Jiang et al., the effect of dendrimer-based NP rigidity on its cellular uptake behaviors was investigated for this study. The designed NPs were mainly based on amine-terminated generation 5 poly(amidoamine) dendrimers (G5·NH_2_), which are highly branched and monodispersed and have a well-defined structure, composition, and geometry [24,25,26,27]. Compared with higher and lower generation dendrimers (e.g., G6 and G4), G5·NH_2_ dendrimers with relatively lost cost display more desirable spherical structure for acting as stabilizers for the inner-formed metal NPs. By harnessing their abundant inner cavity, hard metal NPs could be encapsulated as rigidity regulators. When forming dendrimer/metal nanocomposites, two distinct structures could be formed [28,29,30]: the former comprises a small metal NP confined within the internal cavity of a single dendrimer molecule; the latter comprises a large metal NP surrounded by multi-dendrimers, wherein the number of dendrimers per NP may vary. Considering the structural certainty, the former was preferred in this work for desirable rigidity tuning.

Until now, abundant research related to the usage of dendrimers and dendrimer-based NPs in biomedical applications has been reported [31,32,33]. Some studies have involved their interactions with cells. For example, it was reported that the internalized dendrimer/Au NPs were located in lysosomes [30,34]. In addition, their cellular behaviors could be enhanced by targeting ligand modification [35,36,37]. However, little is known regarding the effect of the rigidity of dendrimers on their cellular uptake behaviors.

**Figure 1:**
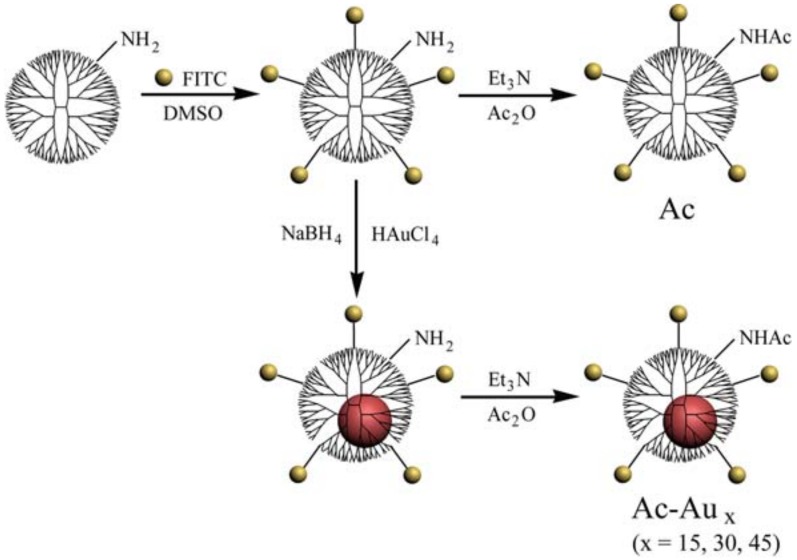
Schematic illustration of the synthesis of Ac and Ac-Au_x_ dendrimers.

Herein, NPs based on G5·NH_2_ dendrimers with similar physicochemical properties but distinct rigidity were produced as models to study their potential rigidity-dependent cellular uptake behaviors. G5·NH_2_ dendrimers were first labeled with fluorescein isothiocyanate (FITC) for fluorescence microscopic analysis. The dendrimer rigidity was regulated by forming gold NPs with tunable size in their internal cavity by simply adjusting the dendrimer/gold salt feed molar ratio. Thereafter, their terminal amines were acetylated to neutralize their surface charge. The preparation process is illustrated in Figure 1. The formed NPs were characterized to determine their size, surface potential, and fluorescence intensity. Their cellular uptake behaviors and intracellular localization were assessed via flow cytometry and confocal microscopic analyses, respectively.

## 2. Materials and Methods

### 2.1. Materials

Amine-terminated generation 5 poly(amidoamine) dendrimer was purchased from Dendritech (Midland, MI, USA). Chloroauric acid (HAuCl_4_) was purchased from Sinopharm Chemical Reagent Co., Ltd. (Shanghai, China). FITC, acetic anhydride (Ac_2_O), and triethylamine (Et_3_N) was obtained from J&K Chemical Reagent Co., Ltd. (Beijing, China). Dimethyl sulfoxide (DMSO) was provided by Greagent. Sodium borohydride (NaBH_4_) was purchased from Kelong Reagent Co., Ltd. (Chongqing, China). All the chemicals were used without further purification. Dialysis bags with molecular weight cut-off (MWCO) of 14,000 Da were obtained from Shanghai Yuanye Biotechnology Corporation (Shanghai, China). U87MG cells (a human glioblastoma cell line) and L929 cells (a mouse fibroblast cell line) were obtained from the Institute of Biochemistry and Cell Biology, the Chinese Academy of Sciences (Shanghai, China). Minimum essential medium (MEM), trypsin containing EDTA, penicillin-streptomycin solution, and fetal bovine serum (FBS) were purchased from ThermoFisher Scientific (Waltham, MA, USA). Solutions of CCK-8, Hoechst 3334, Lyso-Tracker Red were obtained from Beyotime (Shanghai, China). De-ionized (DI) water (18.2 MΩ·cm) from a water purification system (Synergy, Millipore, MA, USA) was used in all the preparation processes.

### 2.2. Preparation of G5·NH_2_-FITC

The synthesis method was in accordance with the references [38,39]. Briefly, FITC solution was mixed with G5·NH_2_ solution at a molar ratio at 6/1, followed by 24 h magnetic stirring. Then, the crude products were dialyzed and lyophilized to obtain the G5·NH_2_-FITC dendrimers.

### 2.3. Preparation of Acetylated Fluorescent Dendrimers with Distinct Rigidity

Gold NPs were used as regulators to modify the rigidity of the obtained G5·NH_2_-FITC dendrimers. Using sodium borohydride reduction chemistry [34,40], gold NPs were formed with kinds of dendrimer/gold salt molar ratio, such as 1/15, 1/30, 1/45. Thereafter, the remaining terminal amines of the raw product were acetylated, as reported previously [41,42]. The crude product was dialyzed and lyophilized to obtain the final Ac-Au_x_ (x = 15, 30, 45) dendrimers. Furthermore, acetylated fluorescent dendrimers without a gold NP core were generated, which were denoted as Ac.

### 2.4. Characterization Techniques

Proton nuclear magnetic resonance (^1^H NMR) spectra of the formed dendrimers were acquired using a Bruker AV 300 NMR (Karlsruhe, Germany) spectrometer in D_2_O solvent. Their UV–visible (UV-Vis) absorption spectra were measured using a UV spectrophotometer (UV-1800, Shimadzu, Kyoto, Japan). The size and morphology of the formed Au NPs were assessed via transmission electron microscopy (TEM, 2010F, JEOL, Tokyo, Japan). The hydrodynamic size and surface potential of the formed dendrimers dissolved in pH 7.4 phosphate buffer saline were measured via dynamic light scattering (DLS, Nano ZS90, Malvern, UK). The fluorescence intensities of their aqueous solutions were measured using a fluorescence spectrophotometer (RF-5301PC, Shimadzu, Kyoto, Japan).

### 2.5. Cell Culture

U87MG cells and L929 cells were regularly cultured and sub-cultured in MEM supplemented with 10% FBS and 1% penicillin-streptomycin at 37 °C and 5% CO_2_ in a humidified incubator.

### 2.6. Analysis of Cellular Uptake Behaviors and Intracellular Localization

Flow cytometry analysis was performed to investigate the cellular uptake behaviors of the formed dendrimers. Briefly, 1.0 × 10^5^ cells per well were co-incubated with kinds of dendrimers. After co-incubation for 3 h or 6 h, the cells were collected and analyzed via flow cytometry (NovoCyte, ACEA, San Diego, CA, USA). For each sample, 1 × 10^4^ cell events were measured.

For analysis of intracellular localization, 2 × 10^5^ U87MG cells were co-incubated with kinds of dendrimers at a final concentration of 5.0 μM. After co-incubation for 6 h, the cells were successively incubated with Lyso-Tracker Red and Hoechst33342 to stain the lysosomes and the nucleus in accordance with the manufacturer’s instructions. Multiple laser channels (excitation wavelengths = 455 nm, 519 nm, and 589 nm) were utilized to excite Hoechst 33342, FITC, and Lyso-Tracker Red, respectively. Their fluorescence emissions through three corresponding channels were recorded using confocal laser-scanning microscopy (LSM 780, Carl Zeiss, Oberkochen, Germany).

## 3. Results and Discussion

### 3.1. Preparation and Characterization of Ac-Au_x_ Dendrimers

G5·NH_2_ dendrimers were first fluoresce-labeled using FITC. Successful modification of FITC was confirmed using ^1^H NMR spectrometry (Figure S1), which revealed several new peaks ranging 6–8 ppm after FITC modification [38]. The number of FITC moieties was estimated to be 5.7 on each dendrimer by calculating the corresponding integrated area of G5·NH_2_ and FITC. The successful linkage of FITC was also confirmed via UV-Vis spectrometry (Figure S2). The peak observed at approximately 500 nm could be attributed to the attached FITC moiety. In addition, the solution changed from colorless to a bright orange color after FITC modification.

As rigidity regulators, gold NPs were size-tunable synthesized in the inner cavity of G5·NH_2_-FITC dendrimers via the sodium borohydride reduction method [38]. The size of the formed Au NPs could be regulated by changing the dendrimer/gold salt feed ratio. Finally, acetylation was carried out to neutralize the remaining surface amines of dendrimers, as confirmed by the ^1^H NMR spectra (Figure S3). The spectra for all formed Ac and Ac-Au_x_ (x = 15, 30, 45) displayed proton signals at approximately 1.87 ppm, indicating the existence of –CH_3_ in acetamide groups. The calculated number of their acetamide groups was in agreement with that of dendrimer terminal amines (approximately 110), indicating the success of thorough acetylation [25].

**Figure 2:**
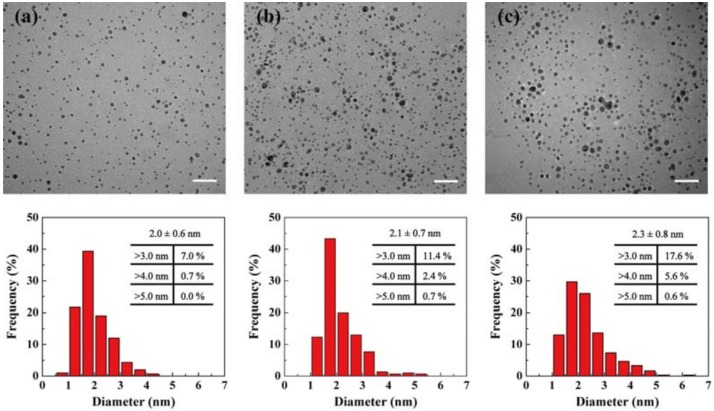
TEM images and the corresponding size distribution histograms of the formed Au NPs in Ac-Au_15_ (**a**), Ac-Au_30_ (**b**), and Ac-Au_45_ (**c**) dendrimers. Scale bars: 20 nm.

The existence of gold NPs was confirmed via UV-vis spectrometry (Figure S4). There was a sharp peak observed at approximately 500 nm, typically attributed to the overlapping of FITC and gold NPs absorbance peak [38]. The color solution changed from yellow to wine red, further indicating the formation of gold NPs (Figure S4, inset). Furthermore, the size of gold NPs was characterized via transmission electron microscopy (TEM) imaging (Figure 2). The gold NPs thus formed were relatively uniform with a fairly narrow size distribution. Their size increased with the gold salt/dendrimer feed molar ratio from 2.0 ± 0.6 nm to 2.3 ± 0.8 nm, which was much smaller than that of G5·NH_2_ dendrimer (5 nm). Further analysis revealed that the percentage of NPs with diameters exceeding 3 nm and 4 nm (60% and 80% of the G5·NH_2_ dendrimer size) increased up to 17.6% and 5.6% for Ac-Au_45_, respectively. These larger Au NPs were more likely to be surrounded by multiple G5 dendrimers. To exclude the effect of NP size, only Ac, Ac-Au_15_ and Ac-Au_30_ were selected to assess their cellular uptake behavior.

**Figure 3:**
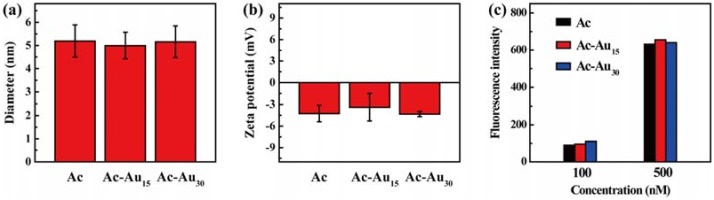
Hydrodynamic size (**a**), surface potential (**b**), and fluorescence intensity (**c**) of the obtained dendrimers.

The hydrodynamic size and surface charge of these gold NPs were measured. All NPs had a similar size of approximately 5 nm with narrow distributions (Figure 3a,b and Figure S5). They all had very weak surface charge, further indicating the success of acetylation. Furthermore, their fluorescence intensities were similar at the same concentrations (Figure 3c). These data confirmed that Ac, Ac-Au_15_, and Ac-Au_30_ displayed similar hydrodynamic size, surface charge, and fluorescence property. The only difference in their rigidity makes them suitable models for analysis of their potential rigidity-dependent cellular uptake behavior.

### 3.2. Analysis of Cellular Uptake Behaviors and Intracellular Localization

**Figure 4:**
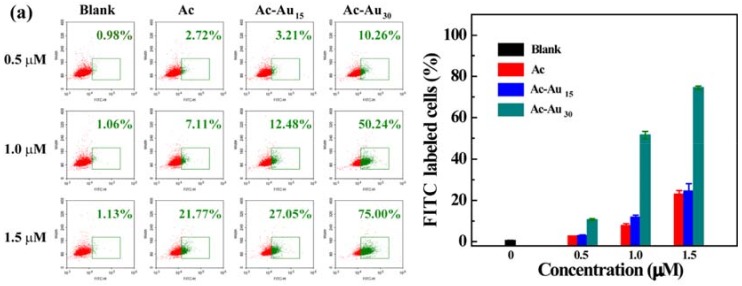

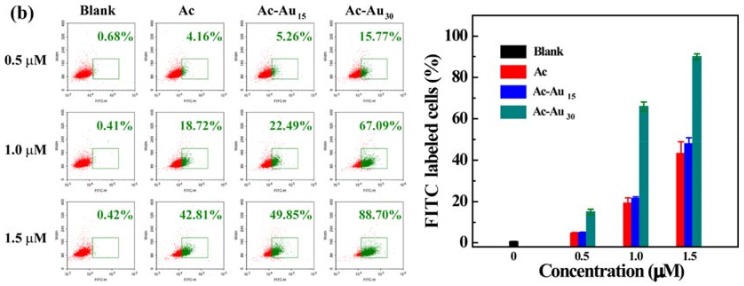
Flow cytometry analysis of U87MG cells after treatment with Ac, Ac-Au_15_, Ac-Au_30_ dendrimers for 3 h (**a**) and 6 h (**b**).

To study the cellular uptake process more clearly, two kinds of normally used cell lines with distinct properties were employed as models. One model used U87MG cells, a kind of cancer cells owning different surface proteins from normal cells. The other used L929 cells, which are a kind of normal cells. Using the U87MG cells as a model system, the effect of NP rigidity on their cellular uptake behaviors was quantitatively analyzed using flow cytometry. After a 3 h co-incubation with the same concentration of different dendrimers (Figure 4a), the percentages of FITC-labeled cells increased with an increase in NP rigidity. When treated with Ac-Au_30_ at 1.5 μM, the percentage of FITC-positive cells was 74.4%, which was markedly higher than that of Ac (23.0%) and Ac-Au_15_ (24.5%). This suggested a more rapid internalization process for Ac-Au_30_. Furthermore, their cellular uptake behaviors were concentration-dependent. For Ac-Au_15_, the percentage of FITC-positive cells increased from 7.77% and 11.83% to 51.53% with NP concentration increasing from 0.5 and 1.0 to 1.5 μM. The cellular uptake efficiency also depended on incubation time (Figure 4b). After 6 h of co-incubation, the percentage of FITC-positive cells increased markedly at all concentrations of the produced NPs. Moreover, a similar phenomenon was observed when using L929 cells as a model system after 3 h and 6 h of co-incubation (Figure S6), further verifying their rigidity-dependent cellular uptake behaviors. This indicated that their cellular uptake behaviors mainly depended on their rigidity, showing no cell line specificity in the studied conditions. Although the inner formed Au NPs cannot interplay with cells directly, they can change the interaction between NPs and cells by altering the flexibility of the NPs. In the process of cellular internalization, different deformation degrees of NP occurred. Less degree of deformation facilitated the cellular uptake [23].

**Figure 5:**
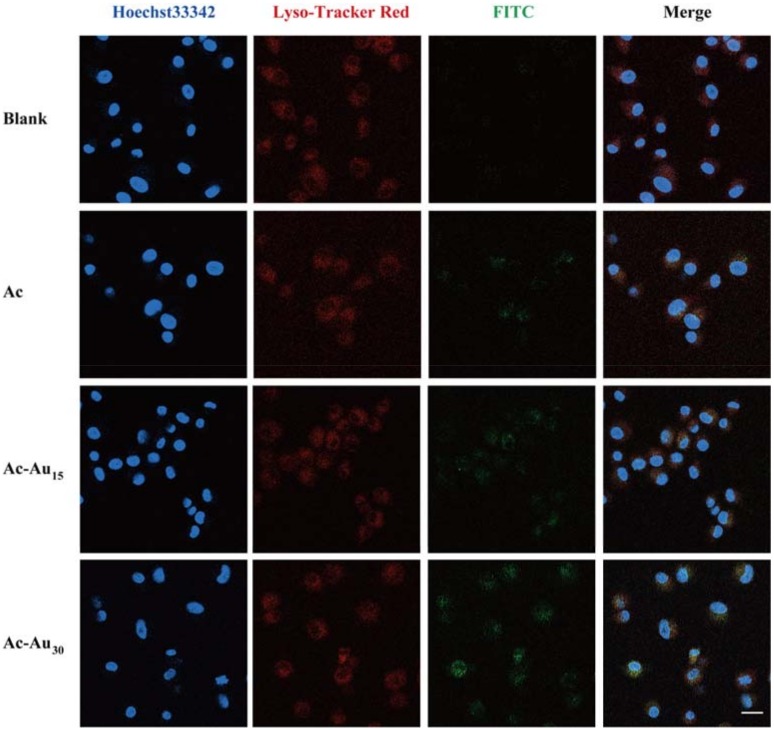
Confocal fluorescence microscopic images of U87MG cells after 6 h of co-incubation with the formed dendrimers. The fluorescence of Hoechst 33342, Lyso-Tracker Red and FITC were pseudo-labeled with blue, red, and green fluorescence, respectively. Scale bars: 20 μm.

Furthermore, intracellular localization of the formed NPs was assessed via confocal laser-scanning microscopy (Figure 5), which revealed nucleus (Hoechst 33342), lysosomal (Lyso-Tracker Red), and the FITC-labeled dendrimers. The imaging results revealed that most of the internalized NPs surrounded the cell nuclei, thereby implying cytoplasmic localization. The increasing fluorescence intensity of FITC from Ac, Ac-Au_15_ to Ac-Au_30_ also indicated the enhanced cellular uptake with the increasing of Au NP size, which was constant with the flow cytometry data.

## 4. Conclusions

In summary, G5 dendrimers were selected as a model system to investigate the effect of NP rigidity on their cellular uptake behaviors. The dendrimers were fluorescence-labeled with FITC and their surface charges were shielded through acetylation. By forming size-tunable Au NPs in the inner cavity of the dendrimers, the rigidity of the final NPs could be effectively regulated. The formed NPs had similar hydrodynamic size, surface charge, and fluorescence intensity at the same molar concentration. Flow cytometry analysis revealed that dendrimers with higher gold content displayed faster cellular uptake. Confocal microscopic images revealed that the internalized NPs were more likely to localize in the cytoplasm. The present findings may potentially inspire new strategies to prepare NPs with customizable rigidity for biomedical applications, such as in vivo blood pool imaging, tumor imaging, and in vivo drug delivery.

## Supplementary Materials

The following are available online at http://www.mdpi.com/1999-4923/10/3/99/s1, Figure S1: ^1^H NMR spectra of G5·NH_2_ (a) and G5·NH_2_-FITC (b), Figure S2: UV-vis spectra of G5·NH_2_ (a) and G5·NH_2_-FITC (b). The insets show their corresponding aqueous solutions, Figure S3: ^1^H NMR spectra of Ac (a), Ac-Au_15_ (b), Ac-Au_30_ (c), and Ac-Au_45_ (d), Figure S4: UV-vis spectra of Ac (a), Ac-Au_15_ (b), Ac-Au_30_ (c), and Ac-Au_45_ (d). The insets show their corresponding aqueous solutions, Figure S5: Hydrodynamic size and polydispersity of the formed Ac (a), Ac-Au_15_ (b), and Ac-Au_30_ (c), Figure S6: Flow cytometry analysis of L929 cells after treatment with Ac, Ac-Au_15_, Ac-Au_30_ for 3 h (a) and 6 h (b).
