# Supplementary material for: Cellular Uptake Behaviors of Rigidity-Tunable Dendrimers
Source: Pharmaceutics. 2018 Jul 19;10(3):99. doi: 10.3390/pharmaceutics10030099 (PMC6161299; doi:10.3390/pharmaceutics10030099)
Supplement: Supplementary file 1 [file pharmaceutics-10-00099-s001.pdf]

Supplementary Materials: Cellular uptake behaviors of rigidity-tunable dendrimers

Hui Liu, Jingjing Wang, Wenchao Li, Jie Hu, Min Wang and Yuejun Kang

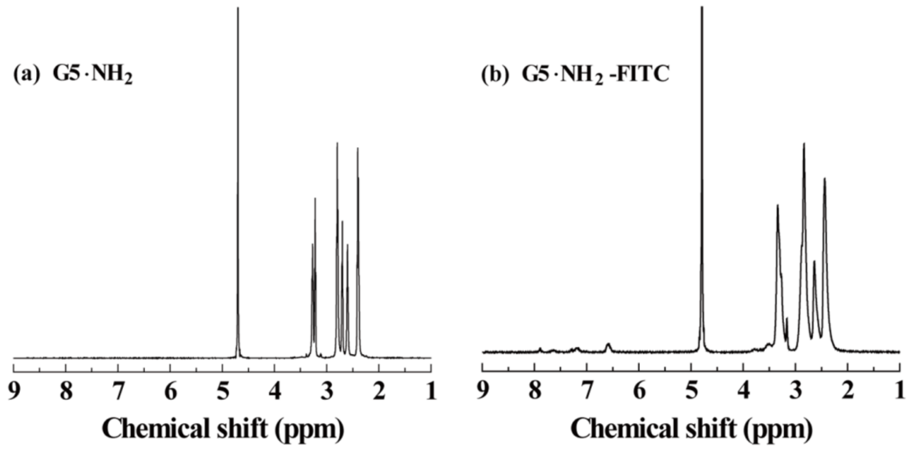

Figure S1. ^{1}H NMR spectra of G5·NH_2 (**a**) and G5·NH_2-FITC (**b**) dendrimers.

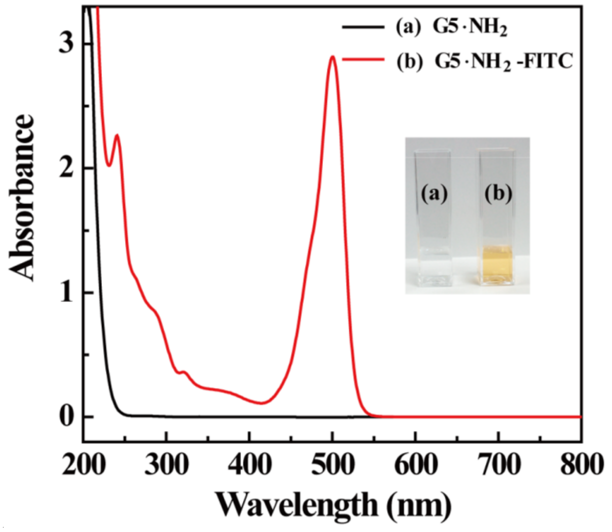

Figure S2. UV-vis spectra of G5·NH_2 (**a**) and G5·NH_2-FITC (**b**) dendrimers. The insets show their corresponding aqueous solutions.

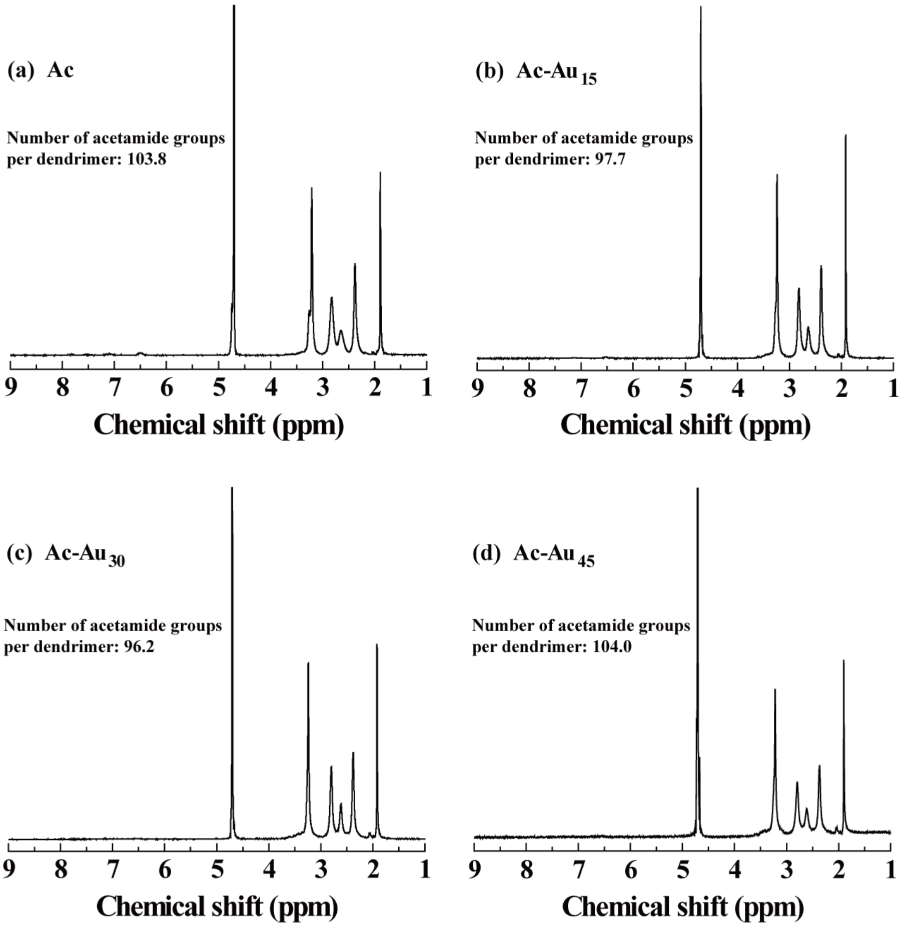

Figure S3. ^{1}H NMR spectra of Ac (**a**), Ac-Au$_{15}$ (**b**), Ac-Au$_{30}$ (**c**), and Ac-Au$_{45}$ (**d**) dendrimers.

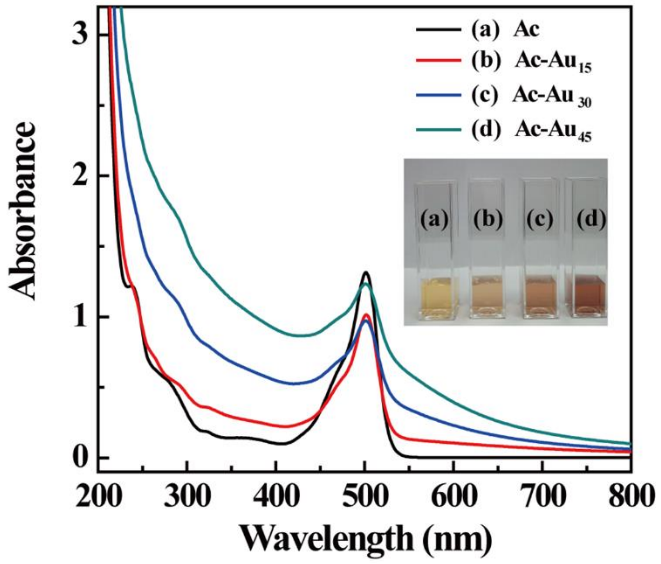

Figure S4. UV-vis spectra of Ac (**a**), Ac-Au$_{15}$ (**b**), Ac-Au$_{30}$ (**c**), and Ac-Au$_{45}$ (**d**) dendrimers. The insets show their corresponding aqueous solutions.

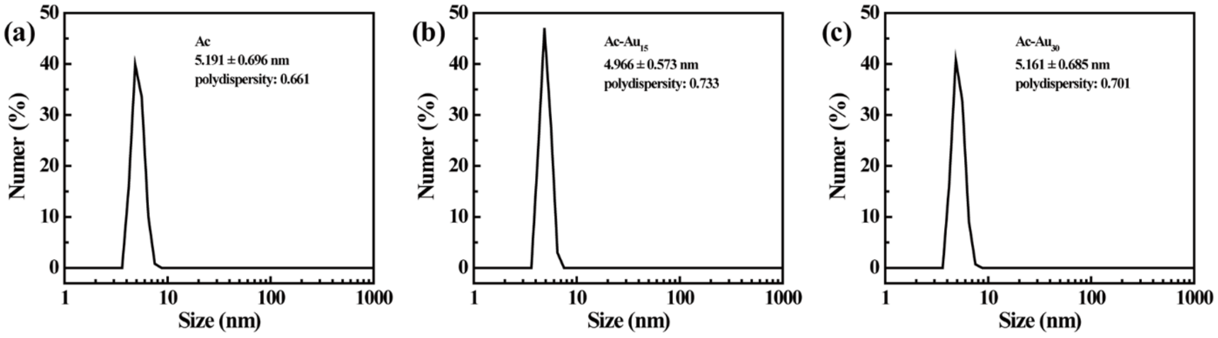

Figure S5. Hydrodynamic sizes and polydispersities of the formed Ac (a), Ac-Au$_{15}$ (b), and Ac-Au$_{30}$ (c) dendrimers.

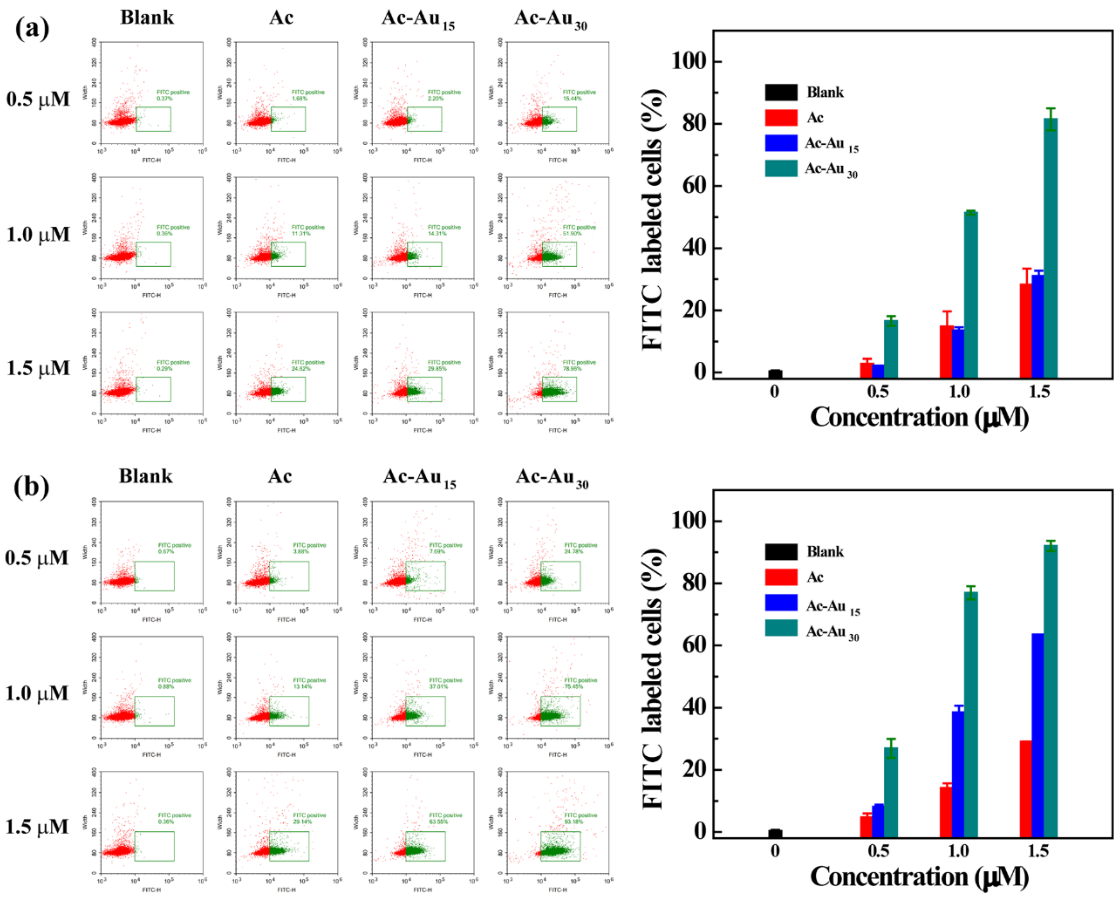

Figure S6. Flow cytometry analysis of L929 cells after treatment with Ac, Ac-Au$_{15}$, Ac-Au$_{30}$ for 3 h (**a**) and 6 h (**b**).
